# Effect of Dietary Betaine on Muscle Protein Deposition, Nucleic Acid and Amino Acid Contents, and Proteomes of Broilers

**DOI:** 10.3390/ani12060736

**Published:** 2022-03-15

**Authors:** Rui Chen, Yuduo Song, Mi Yang, Chao Wen, Qiang Liu, Su Zhuang, Yanmin Zhou

**Affiliations:** College of Animal Science and Technology, Nanjing Agricultural University, Nanjing 210095, China; 2018205030@njau.edu.cn (R.C.); 2019105082@stu.njau.edu.cn (Y.S.); 2019105081@njau.edu.cn (M.Y.); wenage@163.com (C.W.); liuayang@njau.edu.cn (Q.L.)

**Keywords:** betaine, breast muscle, nucleic acid, amino acid, proteomic, broiler

## Abstract

**Simple Summary:**

In animal production, growth trials have shown that betaine has a positive effect on growth performance, especially improving meat yield. Proteins are the largest unit comprising muscle cells except moisture. However, how betaine regulates muscle protein anabolism and abundance levels remains unclear. Therefore, this study was conducted to investigate the effect of dietary betaine on growth performance, muscle protein deposition, and proteome of broilers. The results suggested that betaine could improve growth performance and muscle protein deposition of broilers. Alterations in muscle nucleic acids, amino acids, and protein abundance levels were involved in this process. Proteomic analysis further revealed that 35 proteins in breast muscle were identified as differentially abundant proteins by betaine supplementation, which were mainly related to cell differentiation, small molecule metabolic process, and tissue development.

**Abstract:**

To investigate the effect of betaine supplementation on growth performance, muscle protein deposition, muscle nucleic acid and amino acid contents, and muscle proteome of broilers, 160 one-day-old male partridge shank broiler chickens were randomly divided into 2 groups with 8 replicates of 10 broilers each. Broilers were fed a basal diet alone, or a basal diet supplemented with 1000 mg/kg betaine. Compared with the control group, the betaine group significantly increased (*p* < 0.05) the broilers average daily gain, the levels of serum insulin-like growth factor-1 (IGF-1), growth hormone (GH), total protein (TP), the contents of muscle absolute protein deposition, RNA, Ser, Glu, Met, and Phe, and the ratio of RNA/DNA, and decreased (*p* < 0.05) the feed conversion ratio and serum blood urea nitrogen content. Moreover, proteomic analysis revealed 35 differentially abundant proteins (DAPs) in the betaine group compared with the control group, including 27 upregulated proteins and 8 downregulated proteins (*p* < 0.05). These DAPs were mainly related to cell differentiation, small molecule metabolic process, and tissue development. In conclusion, diets supplemented with 1000 mg/kg betaine improved growth performance and muscle protein deposition of broilers. Increased serum GH, IGF-1, and TP contents, and alterations in muscle nucleic acids, amino acids, and protein abundance levels were involved in this process.

## 1. Introduction

Betaine is a trimethyl derivative of the amino acid glycine, which is widely distributed in nature [1]. Due to its chemical structure, betaine plays an important role in osmotic protection and transmethylation. As an organic osmolyte, betaine can maintain cell structure, and protect protein and enzyme activities against environmental stress. As a methyl donor, betaine can take part in the methionine cycle and donates its labile methyl group to synthesize numerous substances, such as carnitine, choline, and creatine [1,2]. In animal production, growth trials revealed the positive effects of betaine on growth performance, especially increasing meat yield [3,4,5,6]. In human nutrition, betaine has been used as an ergogenic aid to improve muscle endurance, strength, and power performance [7,8,9]. It had been confirmed that supplemental betaine could increase myotubes size and promote myofibers differentiation in C2C12 myoblasts, and enhance skeletal myogenesis of mice [10,11]. Increasing evidence has indicated that the growth and function of skeletal muscle are closely related to betaine. Our laboratory recently reported that betaine could improve muscle growth of broilers via altering myogenic genes expression and insulin-like growth factor-1 (IGF-1) signaling pathway [12]. Proteins are the largest unit of muscle cells except moisture. However, how betaine regulates muscle protein anabolism, abundance levels, and function remains unclear.

Translation, occurring in the cytoplasm, is a process through which proteins are synthesized, including DNA transcription into RNA, RNA translation into peptide chain, and peptide chain processing into protein [13]. In this process, the quantity of DNA was believed to be normally stable but the content of RNA, primarily associated with ribosomes, was closely related to the rate of protein synthesis [14]. Amino acids are the basic components of proteins and one of the most important components in nitrogen cycle, which determines the structure and function of proteins [15]. Therefore, nucleic acids and amino acids play vital roles in muscle protein anabolism. Moreover, proteomic is now one of the most important methods for identifying and comparing changes in protein abundance levels caused by development-, disease-, stress-, and treatment-related changes [16]. With the development of labeling method and mass spectrometry, isobaric tags for relative and resolute quantification (iTRAQ) coupled with liquid chromatography tandem mass spectrometry (LC-MS/MS) has been widely used for protein abundance analysis and functional protein identification, which is more reliable than the traditional two-dimensional electrophoresis due to its high throughput and stability [17,18]. Recently, more researchers have obtained substantial information about functional proteins, using iTRAQ technology, that played critical roles in muscle growth and meat quality under stress or nutritional strategies [18,19,20]. However, to our knowledge, a proteomic study in the muscle tissue of broilers affected by betaine has not been conducted. Therefore, our study was designed to investigate the effect of betaine on muscle protein deposition, nucleic acid and amino acid contents, and used the iTRAQ technology to identify the functional proteins affecting muscle growth and function of broilers.

## 2. Materials and Methods

### 2.1. Animals, Experimental Design, Diets, and Management

A total of 160 1-day-old male partridge shank broiler chickens with similar birth weights (39.0 ± 0.1 g) were randomly divided into 2 groups, with 10 birds in each group for 8 repetitions, and the feeding experiment was carried out for 52 days. The control group broilers were fed a basal diet (Table 1), and the experimental group birds were fed a basal diet supplemented with 1000 mg/kg anhydrous betaine (96%), which was obtained from Yixing Skystone Feed Co., Ltd. (Yixing, Jiangsu, China). The level of betaine was selected according to our previous study that 1000 mg/kg anhydrous betaine could improve growth performance and breast muscle yield of broilers [12]. Birds were allowed free access to mash feed and clean water under a 23 h light/1 h dark lighting program. The ambient temperature of experimental room was maintained at 33 °C for the first 3 days, then slowly decreased by 3 °C each week to a final temperature of 20 °C. At 52 days of age, broilers were weighed after a 12 h feed deprivation, and feed intake was recorded by replicate to calculate average daily gain (ADG), average daily feed intake (ADFI), and feed conversion ratio (FCR).

### 2.2. Sample Collection

At 52 days of age, one broiler from each replicate (cage) was randomly selected, weighed, and euthanized by cervical dislocation. Blood samples were collected from wing veins and centrifuged at 3000× *g* for 15 min at 4 °C to separate serum, which were refrigerated at −20 °C until determination. Then, whole breast muscles were excised, weighed, and a part of samples collected from pectoralis major muscles were immediately stored at −80 °C for further analysis.

### 2.3. Serum Parameters Determination

The concentrations of serum growth hormone (GH), triiodothyronine (T3), tetraiodothyronine (T4), insulin (INS), and IGF-1 were determined using chicken-specific enzyme-linked immunosorbent assay (ELISA) kits, and the contents of serum total protein (TP), glucose (GLU), globulin (GLB), albumin (ALB), ammonia (NH3), and blood urea nitrogen (BUN) were quantified using corresponding chemical kits. All the above kits were purchased from Nanjing Jiancheng Bioengineering Institute (Nanjing, Jiangsu, China), and used as per the manufacturer’s instructions.

### 2.4. Muscle Protein Deposition and Nucleic Acids Contents Measurement

According to the AOAC (2000) method, the moisture and crude protein (CP) components in breast muscle samples were analyzed. Moisture content was determined by weight loss after 12 h in an oven at 105 °C, and CP composition was accurately measured using the Kjeldahl method. Absolute protein deposition was calculated at breast muscle weight × (1 − moisture%) × CP%. Relative protein deposition was calculated as absolute protein deposition divided by broilers weight (g/kg). The concentrations of DNA and RNA in breast muscle were extracted using DNAiso and RNAiso reagents (Takara Biotechnology, Dalian, Liaoning, China), respectively, in accordance with the manufacturer’s instructions. The concentrations of nucleic acids were quantified by ND-1000 spectrophotometer (Nano Drop Technologies, Wilmington, DE, USA).

### 2.5. Muscle Amino Acids Contents Measurement

About 0.2 g of muscle sample was put into a sealed evacuated tube (filled with nitrogen), acidly hydrolyzed with 6 mol/L hydrochloric acid, and heated in an oven at 110 °C for 22 h. After cooling, the volume was made up to 50 mL with ultrapure water. Then, 1 mL of sample was evaporated to dryness in a rotary evaporator at 60 °C, and then 2 mL of hydrochloric acid solution (0.02 mol/L) was added to reconstitute it. After filtering with 0.22 μm disposable filter paper, the amino acid contents in the test samples were analyzed by an automatic amino acid analyzer (L-8900; Hitachi, Tokyo, Japan).

### 2.6. iTRAQ Experiments

In order to reduce individual differences, identical pieces of muscle tissue from 2 birds in the same group were combined into one biological replicate. Each group received 4 biological replicates. About 0.2 g of muscle samples were ground into powder in liquid nitrogen, and then SDT lysis buffer (4% SDS, 1 Mm DTT, 100 mM Tris-HCI, pH 7.6) was added. After that, samples were heated at 100 °C for 3 min, followed by ultrasonic treatment for 5 min, and then further heated at 100 °C for 3 min. The supernatant was collected by centrifugation at 14,000× *g* for 40 min at 20 °C, and the concentrations of extracted proteins were determined using the BCA protein assay kit (Thermo Scientific, Waltham, MA, USA).

Protein digestion was conducted through the FASP procedure [21]. Protein samples at 300 μg were added to dithiothreitol for a final concentration of 100 Mm, and heated at 100 °C for 5 min. After cooling to room temperature, 200 μL UA buffer (8 M Urea, 150 mM Tris-HCl, pH 8.0) was added to the samples and centrifuged, twice, at 12,000× *g* for 15 min in 10 kD ultrafiltration filters. Subsequently, samples were mixed with 100 μL IAA (50 mM iodoacetamide in UA) and incubated at room temperature for 30 min in the dark, followed by centrifugation at 12,000× *g* for 10 min. After that, 100 μL UA buffer was added to the samples and centrifuged at 12,000× *g* for 10 min, twice. Additionally, 100 μL of NH_4_HCO_3_ buffer was added to the filter, followed by centrifugation at 12,000× *g* for 10 min, twice. Next, 40 μL of trypsin (6 μg trypsin in 40 μL NH_4_HCO_3_ buffer) was added to protein suspension at 37 °C for 18 h, followed by centrifugation at 12,000× *g* for 10 min. Finally, the filtrate was collected and the thermo desalting spin column was desalted for peptide quantification.

Each sample containing 100 μg digested protein was labeled with the iTRAQ reagent-8 plex multiplex kit (Applied Biosystems, Grand Island, NY, USA), as per the manufacturer’s instructions. Samples were mixed with equal volume, desalted, and dried by vacuum. The labeled peptides were fractionated using the pierce high pH reversed-phase peptide fractionation kit on a HPLC system (Thermo Scientific). Ten fractions were collected, vacuum dried, and reconstituted with 0.1% formic acid. The Easy nLC-1200 HPLC system (Thermo Scientific) coupled with a Q-Extractive HF-X mass spectrometer (Thermo Scientific) was used for LC-MC/MC analysis. Samples were first injected into a trap C18 column (100 μm × 20 mm, 5 μm) and then passed through an analyzed C18 column (75 μm × 150 mm, 3 μm) for gradient separation at a flow rate of 300 nl/min. The MC data acquisition parameters were set as follows: analysis time, 60 min; detection mode, positive ion; precursor ion scan range, 300–1800 m/z; primary MC resolution, 60,000 at 200 m/z; automatic gain control (AGC) target, 3 × 10^6^; maximum ion accumulation time (MIT), 50 ms. Peptide secondary MC was collected according to the following methods: 20 highest collision energy dissociation fragment files collected per full scan (MS2 scan); resolution, 15,000 at 200 m/z; AGC target, 1 × 10^5^; MIT, 25 ms; activation type, HCD; isolation window, 1.6 m/z; normalized collection energy: 32 eV.

### 2.7. Protein Identification, Quantification, and Bioinformatics Analysis

The raw files were analyzed with MaxQuant software (Version 1.6.0.16 for Windows, Max Planck Institute of Biochemistry, Munich, Germany) and searched for uniport-Gallus gallus (Chicken) [9031]-34937-20200920 (https://www.uniprot.org/; Accessed on 20 September 2020). Searching parameters were set as follows: type, reporter ion MS2; reporter mass tolerance, 0.005 Da; max missed cleavages, 2; peptide tolerance, 10 ppm; MS/MS tolerance, 20 ppm; fixed modifications, carbamidomethyl (C); variable modifications, oxidation (M), acetyl (protein N-term), iTRAQ 8plex (K), iTRAQ 8plex (peptide N-term), deamidation (NQ); false discovery rate (FDR), less than 0.01; protein quantification, unique peptides at least 1. Proteins with a fold change larger than 1.2 or less than 0.83 and *p* < 0.05 (T-tests) were defined as differentially abundant proteins (DAPs). Bioinformatics analysis was performed by Gene Ontology (GO) annotations using Blast2GO software (http://www.blast2go.org; Accessed on 20 September 2020), and the enrichment test was performed by Fisher’s exact test cutoff of 0.05.

### 2.8. Quantitative Real-Time PCR Validation

Total RNA was isolated from muscle samples using RNAiso Reagent (Takara), as per the manufacturer’s instructions. The concentration and purity (OD260/OD280 > 1.8) of RNA were quantified by ND-1000 spectrophotometer (Nano Drop Technologies). Then, all RNA samples were diluted to a uniform concentration with diethyl pyrocarbonate-treated (DEPC) water and reverse transcribed into cDNA with the PrimeScript RT Reagent Kit (Takara). As shown in Table 2, the primer sequence for the test genes were designed using Primer 5.0 software and synthesized by Sangon Biotechnology Co., Ltd. (Shanghai, China). Quantitative real-time PCR was carried out on the ABI StepOnePlus Real-Time PCR System (Applied Biosystems) using the SYBR Premix Ex Taq II Kit (Takara), according to the manufacturer’s protocols. The β-actin and glyceraldehyde 3-phosphate dehydrogena (GAPDH) were used as internal standard to calculate the relative mRNA levels of target genes according to the 2^−ΔΔCT^ method [22]. The mRNA level of each target gene in the broilers fed with the basal diet was assigned a value of one.

### 2.9. Statistical Analysis

Data were analyzed by independent sample T tests using SPSS statistical software (Version 20.0 for Windows, SPSS Inc., Chicago, IL, USA). The differences were considered significant at *p* < 0.05. Results were presented as group means and standard errors of means.

## 3. Results

### 3.1. Growth Performance

As shown in Table 3, diet supplemented with betaine significantly increased (*p* < 0.05) the ADG and decreased (*p* < 0.05) the FCR of broilers from 1 to 52 d of age when compared with the control group. However, a difference in ADFI was not observed between the two groups.

### 3.2. Serum Parameters

Compared with the control group, the concentrations of serum GH, IGF-1, and TP were significantly increased (*p* < 0.05), and BUN was significantly decreased (*p* < 0.05) by dietary betaine supplementation. However, other indicators did not differ between the two groups (Table 4).

### 3.3. Muscle Protein Deposition and Nucleic Acids Contents

Compared with the control group, the contents of absolute protein deposition, RNA, and the ratio of RNA/DNA in breast muscle were significantly increased (*p* < 0.05) by dietary betaine supplementation (Table 5).

### 3.4. Muscle Amino Acids Contents

As shown in Table 6, diet supplemented with betaine significantly increased (*p* < 0.05) the contents of Ser, Glu, Met, and Phe in breast muscle when compared with the control group.

### 3.5. Muscle Protein Identification and Quantification

**Figure 1:**
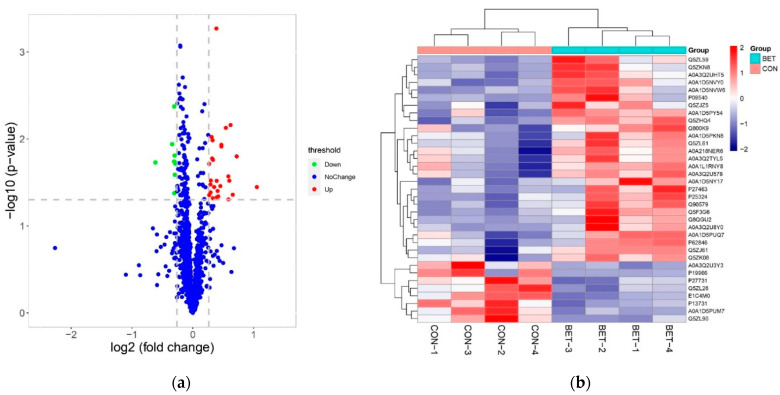
(**a**) Volcano plot of DAPs in breast muscle of broilers fed dietary betaine. Upregulated DAPs (fold change > 1.2 and *p* < 0.05) are shown in red while downregulated DAPs are shown in green (fold change < 0.83 and *p* < 0.05); blue dots represent proteins that are not significantly differentially expressed. (**b**) Hierarchical clustering analysis of the 35 DAPs in breast muscle of broilers detected in control (CON) and betaine (BET) groups.

A total of 1084 proteins were identified and quantified at the FDR of 1% or less through proteomics analysis. Following statistical analysis, 35 proteins were regarded as DAPs (*p* < 0.05) in the betaine group compared with the control group, including 27 upregulated proteins and 8 downregulated proteins (Table 7). The results of the volcano plot and hierarchical cluster analysis for DAPs were shown in Figure 1.

### 3.6. Bioinformatics Analysis

**Figure 2:**
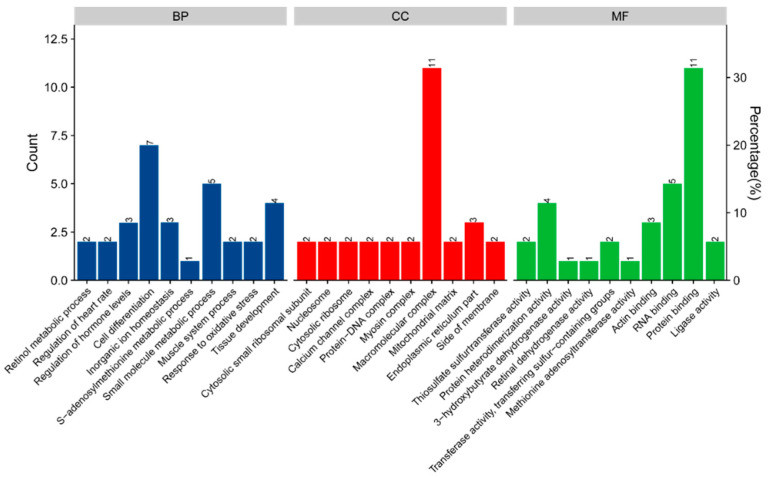
Enrichment of GO analysis of 35 DAPs in breast muscle of broilers fed betaine dietary. BP—biological process; CC—cellular component; MF—molecular function.

The GO enrichment analysis of DAPs was performed to investigate the biological process (BP), cellular component (CC), and molecular function (MF). As shown in Figure 2, the enriched BP category expressed in breast muscle were related to cell differentiation, small molecule metabolic process, tissue development, and inorganic ion homeostasis; macromolecular complex, endoplasmic reticulum part, cytosolic ribosome, and protein−DNA complex were involved in the CC category; and protein binding, RNA binding, protein heterodimerization activity, and actin binding were the most abundant subcategories in the FM category.

### 3.7. Validation of Differentially Abundant Proteins

**Figure 3:**
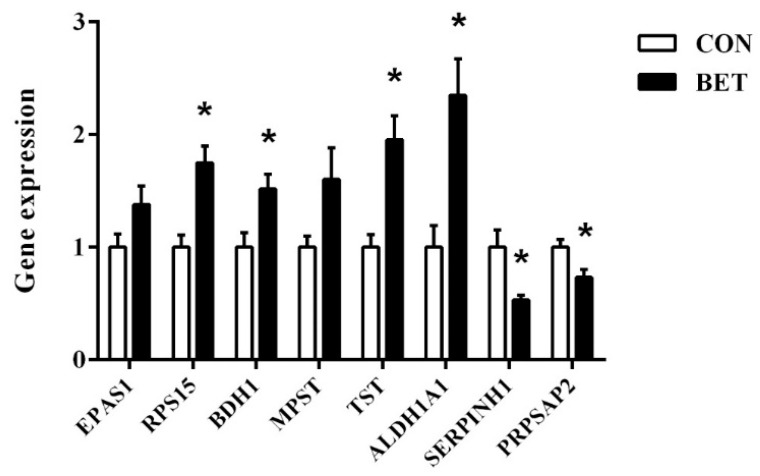
Verified the selected eight DAPs by real-time PCR. CON—control group; BET—betaine group; EPAS1—endothelial PAS domain protein 1; RPS15—ribosomal protein S15; BDH1—3-hydroxybutyrate dehydrogenase 1; MPST—mercaptopyruvate sulfurtransferase; TST—thiosulfate sulfurtransferase; ALDH1A1—aldehyde dehydrogenase 1 family member A1; SERPINH1—serpin family H member 1; PRPSAP2—phosphoribosyl-pyrophosphate-synthetase-associated protein 2. Results presented as means ± SEM (n = 8). * Bars marked with an asterisk were significantly different at *p* < 0.05.

To validate the results obtained from iTRAQ experiments, 8 DAPs were randomly selected for transcript level analysis by real-time PCR. As indicated in Figure 3, the transcript levels of RPS15, BDH1, TST, ALDH1A1, SERP1NH1, and PRPSAP2 showed the same change pattern as their protein levels (*p* < 0.05). There were no significant differences in EPAS1 and MPST at the transcript level, but the variation trends were consistent with the results of iTRAQ experiments.

## 4. Discussion

In animal feed, betaine has long been used as a nutritive additive due to its lipotropic and growth-promoting effects [2]. In this study, the ADG was higher and FCR was lower in the betaine group than those in the control group, which implied that betaine supplementation might have positive effects on growth performance of broilers. This result was similar to the data of Rao et al., who reported that the ADG of broilers was significantly increased by 800 mg/kg betaine supplementation during a 42 d feeding trial [23]. Moreover, some other experiments had also confirmed the growth-promoting effect of betaine in poultry and pigs [5,6,24,25]. This effect might be given by that betaine could improve intestinal health and nutrient digestibility by enhancing intestinal structural integrity and digestive enzyme activity which, in turn, improved the growth performance of broilers [2,5].

Serum biochemical parameters can reflect the nutritional metabolism and health status of animals [26]. GH is a pleiotropic hormone secreted by pituitary gland that coordinates an array of physiological processes, among which the growth-promoting effect on bone and muscle is most notable [27,28]. In the somatotrophic axis, GH can directly act on the cell itself or indirectly activate growth hormone receptor to stimulate IGF-1 synthesis, and then IGF-1 acts on the target cells to promote tissue growth [29,30]. Multiple studies showed that IGF-1 could promote skeletal muscle fibers differentiation and increase myotubes size, resulting in an increase in muscle protein accretion [11,30,31]. It had been reported that supplemental betaine could increase serum GH and IGF-1 levels in pigs and increase IGF-1 concentrations in serum and liver of laying hens [3,32]. Our study also found that the concentrations of GH and IGF-1 in serum of broilers were significantly increased by dietary betaine supplementation, and this might explain why broilers fed with betaine showed a better growth performance in the present study. Moreover, compared with the control group, a higher content of serum TP and a lower BUN content were observed in the betaine group, which were consistent with the previous studies [6,33]. Increased concentration of serum TP was related to the improvement of body protein anabolism [26]. BUN was a key end-product of protein metabolism in the animal body, and decreased serum BUN content implied that more nitrogen was used for protein synthesis [34]. Accordingly, the results of these serum parameters indicated that betaine might play an important role in promoting protein anabolism of broilers.

Skeletal muscle is the main product of protein deposition, which accounts for 35~45% of broilers carcass weight. It is known that proteins are synthesized through a process called translation, which takes place in the cytoplasm and involves DNA transcription into RNA, RNA translation into peptide chain, and peptide chain processing into protein. [13]. In this study, the contents of absolute protein deposition and RNA, and the ratio of RNA/DNA in breast muscle were higher in the betaine group than that in the control group. It was believed that the quantity of DNA in muscle cell was normally stable, but the content of RNA was closely related to the rate of protein synthesis [14]. Moreover, RNA/DNA ratio has been proven as a useful indicator of the nutritional status and growth of animals in relation to protein metabolism [14,35]. Therefore, these results further confirmed the promoting effect of betaine on protein synthesis. We speculated that betaine regulated muscle nucleic acids contents in two aspects. On the one hand, betaine could promote the secretion of IGF-1 to enhance the activity of RNA polymerase, thereby promoting DNA transcription into RNA and accelerating protein synthesis. On the other hand, betaine, acting as a methyl donor, could methylate RNA to increase its stability and prevent degradation. For example, promoting guanylate methyl reaction was conducive to the generation of 5′ end cap of mRNA [13]. Amino acids are the basic components of peptide and protein. Betaine, a methyl donor, can participate in the regeneration cycle of Met, and then decompose into some secondary metabolites, such as Ser and Gly [36]. This may explain the increased Met and Ser contents in breast muscle of broilers fed betaine. Similar results were reported by previous studies that betaine supplementation could increase serum Ser and Met contents of goats, and linearly enhance the content of Met, Ser, and Glu in muscle tissue of ducks [37,38]. However, the reason why the contents of Glu and Phe were increased by betaine supplementation still needs further research. The changes of amino acids compositions will affect the structure and functional expression of protein. Thus, we speculate that betaine may affect the abundance levels and function of skeletal muscle protein.

The iTRAQ technology combined with LC-MS/MS is now one of the most sensitive methods used for quantitative analysis of proteomes. In this study, a total of 1084 proteins were detected through proteomics analysis, in which 35 proteins were identified as DAPs in the betaine group compared with the control group, including 27 upregulated proteins and 8 downregulated proteins. These DAPs were mainly involved in cell differentiation, small molecule metabolic process, tissue development, inorganic ion homeostasis, and S-adenosylmethionine metabolic process. S-AdoMet_synt_C-domain-containing protein, as a methionine adenosyltransferase, participates in the formation of S-adenosylmethionine (SAM), which plays a vital role in transmethylation reactions and the transsulfuration pathway [39]. Thiosulfate sulfurtransferase (TST) and uncharacterized protein (MPST) are involved in transferring sulfur-containing groups. It had been reported that betaine could take part in the metabolism of sulfur-containing amino acids and DNA methylation [36,40]. DNA methylation was known to have regulatory effects on DNA transcription and chromosome structure, which might alter protein functions [41]. Our results indicated that betaine increased muscle Met content and regulated DNA methylation might be related to upregulated S-AdoMet_synt_C-domain-containing protein, TST, and MPST. Transcription regulation plays an important role in muscle growth and function. Phenylalanyl-tRNA synthetase beta subunit (FARSB) and asparagine-tRNA ligase (RCJMB04_13p14) are responsible for attaching L-phenylalanine and L-asparagine to the terminal adenosine of the appropriate tRNA, respectively, to take part in RNA binding and protein biosynthesis. PHD finger protein 20-like protein 1 (PHF20L1) regulate the transcription by RNA polymerase II, and 40 S ribosomal protein S15 (RPS15) is a key component in the assembly of the small ribosomal subunit, which is essential to RNA binding [42]. The upregulation of FARSB, RCJMB04_13p14, PHF20L1, and RPS15 indicated that betaine might play a positive role in RNA binding, which was consistent with the result that the content of RNA in breast muscle was increased by betaine supplementation in this study. Moreover, uncharacterized protein (LOC107050760) positive regulates protein localization to plasma membrane and plays an important role in skeletal muscle contraction and fiber development. Previous studies had reported that betaine could promote muscle growth of animals [4,10,12]. Our results further confirmed that betaine could affect the expression of some functional proteins in muscle. However, from the results of muscle proteomics, the number of DAPs affected by dietary betaine was not much, especially only 6 proteins were upregulated with fold change >1.5. This may be related to low abundance of many transport and signaling proteins which are below detection under the current experimental settings. It could be possible that many DAPs are regulated by post-translational modifications. So, future studies that multiple fractionation scheme and post-translation modifications (PTMs) studies, such as methylation, phosphorylation, and acetylation, are needed to provide comprehensive understanding of the effect of dietary betaine on muscle protein anabolism.

In order to verify the results obtained from iTRAQ experiments, 8 DAPs were randomly selected for transcript level analysis by real-time PCR. Our results showed that the mRNA levels of RPS15, BDH1, TST, ALDH1A1, SERP1NH1, and PRPSAP2 had the same change pattern as their protein levels, supporting the proteomic data. However, there were no significant differences in EPAS1 and MPST at the transcript level, which might be due to the RNA editing, post-translation modification, or other regulatory mechanisms.

## 5. Conclusions

In summary, diets supplemented with 1000 mg/kg betaine improved growth performance and muscle protein deposition of broilers, which were associated with increased serum GH, IGF-1, and TP contents, and alterations in muscle nucleic acids (RNA), amino acids (Ser, Glu, Met, and Phe), and protein abundance levels (27 upregulated proteins and 8 downregulated proteins).

## Figures and Tables

**Table 1:** Composition and nutrient contents of basal diets (as-fed basis).

Items	1–21 Days	22–42 Days	43–52 Days
Ingredients (g/kg)			
Corn	565	612	659
Soybean meal	322	260	234
Corn gluten meal	37.0	42.0	20.0
Soybean oil	26.1	38.7	39.7
Dicalcium phosphate	20.0	16.0	16.0
Limestone	12.0	14.0	14.0
Premix ^1^	10.0	10.0	10.0
L-Lysine·HCl	3.40	3.50	3.50
Sodium chloride	3.00	3.00	3.00
DL-Methionine	1.50	0.80	0.80
Calculated nutrient contents			
Metabolizable energy (MJ/kg)	12.4	13.0	13.1
Crude protein (g/kg)	218	197	177
Lysine (g/kg)	12.3	11.0	10.3
Calcium (g/kg)	10.0	9.6	9.5
Total sulfur amino acids (g/kg)	8.70	7.50	6.80
Methionine (g/kg)	5.10	4.20	3.80
Available phosphorus (g/kg)	4.60	3.90	3.90

^1^ Each kilogram of the diet contained: vitamin A, 10,000 IU; vitamin D_3_, 3000 IU; vitamin E, 30 IU; choline chloride, 600 mg; nicotinamide, 40 mg; calcium pantothenate, 10 mg; riboflavin, 8 mg; pyridoxine·HCl, 4 mg; thiamin, 2.2 mg; menadione, 1.3 mg; folic acid, 1 mg; biotin, 0.04 mg; vitamin B12, 0.013 mg; Mn, 110 mg; Fe, 80 mg; Zn, 65 mg; Cu, 8 mg; I, 1.1 mg; and Se, 0.3 mg.

**Table 2:** Sequences used for real-time PCR primers.

Genes ^1^	GeneBank ID	Primer Sequence, Sense/Antisense	Product Size (bp)
EPAS1	NM_204807.2	TTGACGATGAGCAGTGCCTTTGAG	116
		CCAGGTGTTGGAGCCAGTTGTG	
RPS15	NM_205462.1	ACAACGGCAAGACCTTCAACCAG	86
		CGGCTTGTAGGTGATGGAGAACTC	
BDH1	NM_001006547.2	GGGTCGTGTAGTGAACATCAGTAGC	111
		TACCGCAGGCAGTCAGAGAAGG	
MPST	NM_001277377.1	CTGAAGAACTGGCTGCGAGAAGG	150
		CACGACTTGGAAGCGATGGGAATC	
TST	NM_001167731.1	GATGGCTCCTGGTCTGAATGGTTC	110
		GGCTACAGATACGCTAAGGGACAAC	
ALDH1A1	NM_204577.4	TGGATTGACATGGAGGTGAGAGAGG	100
		AGCCATTGCACGTACCACTCATTC	
SERPINH1	NM_205291.1	GCCGAGAGGAGATGAGGAACCC	106
		ACGAGCCTGCCAATGAAGAGAATG	
PRPSAP2	NM_001006165.1	CATGGTCTGCTATCTTCGGATGCTC	103
		ACTGGAGTTTCTGGATTTCGTGTGG	
β-actin	NM_205518	TGCTGTGTTCCCATCTATCG	150
		TTGGTGACAATACCGTGTTCA	
GAPDH	NM_204305	AGAACATCATCCCAGCGTCC	133
		CGGCAGGTCAGGTCAACAAC	

^1^ EPAS1—endothelial PAS domain protein 1; RPS15—ribosomal protein S15; BDH1—3-hydroxybutyr ate dehydrogenase 1; MPST—mercaptopyruvate sulfurtransferase; TST—thiosulfate sulfurtransferase; ALDH1A1—aldehyde dehydrogenase 1 family member A1; SERPINH1—serpin family H member 1; PRPSAP2—phosphoribosyl pyrophosphate synthetase associated protein 2; GAPDH—glyceraldehyde 3-phosphate dehydrogenase.

**Table 3:** Effect of betaine on growth performance of broilers from 1 to 52 d of age.

Items ^1^	Control	Betaine	*p*-Value
ADG (g)	37.15 ± 0.38	38.51 ± 0.39 *	0.025
ADFI (g)	81.58 ± 0.76	82.35 ± 0.87	0.509
FCR (g/g)	2.20 ± 0.02	2.14 ± 0.02 *	0.040

^1^ ADG—average daily gain; ADFI—average daily feed intake; FCR—feed conversion ratio (feed: gain). * Mean values within a row with an asterisk differ significantly at *p* < 0.05.

**Table 4:** Effect of betaine on serum parameters of broilers at 52 d of age.

Items ^1^	Control	Betaine	*p*-Value
GH (ng/mL)	8.77 ± 0.52	9.70 ± 0.91 *	0.024
T3 (ng/mL)	8.42 ± 0.26	9.15 ± 0.23	0.053
T4 (ng/mL)	91.04 ± 3.08	93.71 ± 5.91	0.277
INS (U/mL)	43.00 ± 1.64	44.61 ± 2.06	0.550
IGF-1 (ng/mL)	345.53 ± 6.50	368.94 ± 5.54 *	0.016
TP (g/L)	41.46 ± 1.12	44.62 ± 0.71 *	0.032
ALB (g/L)	14.05 ± 0.95	14.86 ± 0.62	0.061
GLB (g/L)	27.41 ± 1.22	29.75 ± 0.56	0.111
GLU (mmol/L)	13.44 ± 0.20	13.60 ± 0.29	0.649
BUN (mmol/L)	0.97 ± 0.05	0.75 ± 0.05 *	0.013
NH3 (μmol/L)	173.03 ± 4.55	166.48 ± 4.90	0.344

^1^ GH—growth hormone; T3—triiodothyronine; T4—tetraiodothyronine; INS—insulin; IGF-1—insulin-like growth factor-1; TP—total protein; ALB—albumin; GLB—globulin; GLU—glucose; BUN—blood urea nitrogen; NH3—ammonia. * Mean values within a row with an asterisk differ significantly at *p* < 0.05.

**Table 5:** Effect of betaine on breast muscle protein deposition and nucleic acids contents of broilers at 52 d of age.

Items	Control	Betaine	*p*-Value
Absolute protein deposition (g)	60.38 ± 1.96	68.07 ± 2.03 *	0.016
Relative protein deposition (g/kg BW)	27.47 ± 0.47	29.49 ± 0.83	0.053
RNA (ng/mg)	1084.78 ± 24.43	1198.22 ± 34.87 *	0.019
DNA (ng/mg)	722.54 ± 12.56	744.33 ± 13.73	0.261
RNA/DNA	1.50 ± 0.03	1.61 ± 0.04 *	0.036

* Mean values within a row with an asterisk differ significantly at *p* < 0.05.

**Table 6:** Effect of betaine on breast muscle amino acids contents of broilers at 52 d of age (g/kg).

Items	Control	Betaine	*p*-Value
Asp	22.79 ± 0.29	23.72 ± 0.46	0.111
Thr	11.20 ± 0.18	11.59 ± 0.17	0.135
Ser	9.43 ± 0.14	10.06 ± 0.14 *	0.008
Glu	36.08 ± 0.34	38.31 ± 0.67 *	0.010
Gly	10.78 ± 0.12	11.25 ± 0.19	0.053
Ala	14.63 ± 0.21	15.19 ± 0.22	0.087
Cys	2.17 ± 0.14	2.12 ± 0.13	0.796
Val	12.72 ± 0.19	13.27 ± 0.30	0.140
Met	6.31 ± 0.19	6.91 ± 0.17 *	0.036
Ile	11.54 ± 0.15	12.11 ± 0.27	0.082
Leu	20.31 ± 0.14	20.91 ± 0.24	0.053
Tyr	8.74 ± 0.17	9.10 ± 0.16	0.138
Phe	10.34 ± 0.11	10.83 ± 0.21 *	0.047
Lys	22.01 ± 0.30	22.79 ± 0.23	0.057
His	9.43 ± 0.21	9.85 ± 0.21	0.179
Arg	15.56 ± 0.16	15.93 ± 0.18	0.154
Pro	6.85 ± 0.10	7.08 ± 0.13	0.177

* Mean values within a row with an asterisk differ significantly at *p* < 0.05.

**Table 7:** List of DAPs identified by iTRAQ analysis in breast muscle of broilers fed betaine dietary.

Accession	Protein Name	Gene Name	Fold Change	*p*-Value
Q5ZL59	UBC core domain-containing protein	UBE2D3	1.247	0.017
Q5ZKN8	Transaldolase	RCJMB04_9n21	1.368	0.035
A0A3Q2UHT5	S-AdoMet_synt_C domain-containing protein	N/A	1.384	0.012
A0A1D5NVY0	Uncharacterized protein	USMG5	1.458	0.007
A0A1D5NVW6	Myosin heavy chain 1G, skeletal muscle	MYH1G	1.499	0.027
P09540	Myosin light chain, embryonic	N/A	2.074	0.036
Q5ZJZ5	D-beta-hydroxybutyrate dehydrogenase, mitochondrial	BDH1	1.228	0.030
A0A1D5PY54	Uncharacterized protein	LANCL2	1.239	0.009
Q5ZHQ4	Thiolase_N domain-containing protein	RCJMB04_34i5	1.308	0.001
Q800K9	Surfeit locus protein 4	SURF4	1.275	0.036
A0A1D5PKN8	Uncharacterized protein	MPST	1.315	0.047
Q5ZL61	OBG-type G domain-containing protein	RCJMB04_7i14	1.385	0.012
A0A218NER6	Endothelial PAS domain protein 1	EPAS1	1.327	0.040
A0A3Q2TYL5	Uncharacterized protein	N/A	1.206	0.044
A0A1L1RNY8	Histone H2A	H2AFX	1.337	0.046
A0A3Q2U578	Histone H3	N/A	1.205	0.019
A0A1D5NY17	Transmembrane protein 182	TMEM182	1.223	0.033
P27463	Retinal dehydrogenase 1	ALDH1A1	1.502	0.050
P25324	Thiosulfate sulfurtransferase	TST	1.259	0.017
Q90579	Anion exchange protein	N/A	1.649	0.016
Q5F3G6	PHD finger protein 20-like protein 1	PHF20L1	1.538	0.007
Q8QGU2	Peptidylprolyl isomerase	FKBP12.6	1.256	0.048
A0A3Q2U8Y0	Uncharacterized protein	LOC107050760	1.313	0.030
A0A1D5PUQ7	Uncharacterized protein	PFKM	1.516	0.030
P62846	40S ribosomal protein S15	RPS15	1.577	0.044
Q5ZJ61	Phenylalanyl-tRNA synthetase beta subunit	FARSB	1.253	0.010
Q5ZK08	Asparagine-tRNA ligase	RCJMB04_13p14	1.224	0.041
A0A3Q2U3Y3	Calponin	CNN1	0.653	0.019
P19966	Transgelin	TAGLN	0.810	0.042
P27731	Transthyretin	TTR	0.820	0.019
Q5ZL26	Phosphoribosyl pyrophosphate synthase-associated protein 2	PRPSAP2	0.815	0.026
E1C4M0	40S ribosomal protein S2	RPS2	0.809	0.004
P13731	Serpin H1	SERPINH1	0.790	0.011
A0A1D5PUM7	Uncharacterized protein	IGFN1	0.813	0.016
Q5ZL90	Phosducin-domain-containing protein	RCJMB04_7d1	0.809	0.018

## Data Availability

Not applicable.
